# The impact of COVID-19 pandemic on mental burden and quality of life in physicians: Results of an online survey

**DOI:** 10.3389/fpsyt.2023.1068715

**Published:** 2023-04-13

**Authors:** Lea Wetzel, Marie Halfmann, Noah Castioni, Falk Kiefer, Sarah König, Astrid Schmieder, Anne Koopmann

**Affiliations:** ^1^Department of Addictive Behavior and Addiction Medicine, Central Institute of Mental Health (CIMH), Mannheim, Germany; ^2^Feuerlein Centre on Translational Addiction Medicine (FCTS), University of Heidelberg, Mannheim, Germany; ^3^Medical Faculty, Goethe-University, Frankfurt, Germany; ^4^Institute for Medical Teaching and Medical Education Research, University Hospital Würzburg, Würzburg, Germany; ^5^Clinic for Dermatology, Venereology and Allergology, University Hospital Würzburg, Würzburg, Germany

**Keywords:** COVID-19 pandemic, doctors, physicians, mental health, anxiety, depression, quality of life

## Abstract

**Background:**

In previous pan-/epidemics such as the SARS epidemic of 2002/2003, negative effects on the wellbeing and an increase in symptoms of depression and anxiety were observed in doctors due to social isolation and the threat they experienced. Therefore, it is feared that the COVID-19 pandemic will also have a negative impact on the mental health and quality of life of doctors.

**Objective:**

The impact of the COVID-19 pandemic on the mental health of physicians. In particular, on the subjective anxiety and burden, depression and quality of life for the total sample and subsamples (work in COVID-19 units vs. no work in COVID-19 units).

**Materials and methods:**

In an online survey, 107 physicians (23–42 years) were asked about their mental health during the COVID-19 pandemic. In addition to socio-demographic data, pandemic- and work-related data were also included. For example, infection control measures, deployment on COVID-19 wards and the subjective perceived threat posed by the pandemic. The physicians were asked to rate their perceived anxiety and stress, retrospectively, at 7 different points in time during the pandemic. The Hospital Anxiety and Depression Scale (HADS) was used to retrospectively assess symptoms of anxiety and depression before and after the onset of the pandemic. The quality of life of the participants after 2 years of the pandemic was assessed using the WHO Quality of Life (WHOQOL-BREF).

**Results:**

Both subjective anxiety and burden showed wave-like patterns with higher scores in autumn, winter and spring. We observed significant differences between the seven measurement time points for anxiety [Chi^2^(6) = 197.05, *p* < 0.001] as well as for burden [Chi^2^(6) = 106.33, *p* < 0.001]. Symptoms of depression and anxiety increased significantly during the COVID-19 pandemic (*M* = 14.16, SD = 7.83) compared to the pre-pandemic time [*M* = 7.31, SD = 5.14, *t*_(106)_ = −10.67, *p* < 0.001]. Physicians who worked at COVID-19 units showed higher scores in quality of life related to social relationships (*M* = 70.39, SD = 17.69) than physicians not working at COVID-19 units [*M* = 61.44, SD = 24.55, *t*_(90.14)_ = −2.145, *p* = 0.035]. The multi-factorial ANOVA showed that previous psychiatric illness (*p* < 0.001), greater difference in depression scores (*p* = 0.014), higher anxiety scores (*p* = 0.048) and less work experience (*p* = 0.032) led to lower quality of life.

**Conclusion:**

Hospitals should offer specific support, such as supervision, to prevent the development of longer-term psychiatric sequelae likely to lead to sick leave and high costs for the healthcare system.

**Trial registration:**

The study has been registered at the German Clinical Trials Registry (DRKS-ID: DRKS00028984).

## 1. Introduction

The COVID-19 pandemic has led to 581 million infections and 6.41 million deaths since its onset in 2020 [as of August 2022, (1). In addition to the acute somatic symptoms and long-term consequences of the infection (Long COVID Syndrome), the COVID-19 pandemic also led to an immense psychological burden for the population as a whole and in particular, for employees in medical professions (2–4).

Physicians were already a severely psychologically stressed group prior to the COVID-19 pandemic, with 23% of physicians consuming alcohol in a risky manner [compared to general population: 18.1% (5)], with long working hours and surgical professions proving to be a risk factor for dangerous and risky health behaviors (6). The pooled prevalence for depression or depressive symptoms among physicians prior to the COVID-19 pandemic was 28.8% (7), with high between-study heterogeneity [compared to general population: 9.2%; (8)]. The wide variation of the prevalence rates in the different studies highlights the difficulty in accurately determining the prevalence of depression due to differences in survey instruments. Additionally, studies using self-assessment instruments may yield significantly higher point prevalence rates of depression than studies using psychologist diagnoses. There is evidence that German physicians experience significantly more occupational stress than, for example, Australian physicians and tend to use problem-oriented strategies such as planning or active reframing to cope. Whereas, on the other hand, Australian physicians tend to use emotion-oriented coping strategies such as religion, humor, or radical acceptance (9).

Regarding the quality of life of health care workers, a past study prior to the COVID-19 pandemic showed an average quality of life of participants in the quality of life questionnaire (QLQ: mean *T*-score overall quality of life 47.84) with a concurrent mild level of maladaptive stress and strain in the occupational stress inventory (Revised) (OSI-R: mean *T*-score overall occupational stress 63.87) (10). In a German study examining the quality of life of pre-clinical emergency care physicians by means of the WHOQOL-BREF, higher values tended to be found in the domain of physical quality of life than in the psychological domain or the quality of life with regard to social relationships (11).

The COVID-19 pandemic confronted the already vulnerable group of physicians with additional challenges, such as adapting their work processes to very strict infection control measures and dealing with the increased personal risk of infection due to their work.

At the onset of the COVID-19 pandemic in early 2020, physicians showed a prevalence of 11.1% for symptoms of anxiety and 16.9% for symptoms of depression (12). Meta-analyses of point prevalence rates of anxiety among physicians during the COVID-19 pandemic yielded pooled values ranging from 17% [data from 17 countries, including Croatia, Libya, South Korea, and the United States through September 2020 (13)] to 25.8% [global data through 2022 (14)]. Pooled prevalence rates for symptoms of depression ranged between 20,5% (14) respectively 24% among physicians (13) and 43% among frontline healthcare workers (13). Initial European-wide studies suggest that the level of anxiety, depression, or burden among physicians did not differ significantly between the groups who worked in COVID-19 units and those who did not (15). The prevalence rates of symptoms of depression and anxiety among physicians identified in the above mentioned studies show a similar trend to the results of studies researching prevalence rates of mental disorders during past pan-/epidemics, such as the SARS epidemic which ranged from 18% to 57% (14).

After 2 years of living under pandemic conditions with additional challenges and stress factors in the working lives of physicians, with far-reaching restrictions in their private and family lives, long-term negative consequences for the physician's mental health can be feared. Based on the existing current studies, we assume that the surveyed physicians in our study will possibly show a deterioration of their mental health, especially of symptoms of anxiety and depression, compared to before the outbreak of the pandemic.

As shown above, a relatively high prevalence rate of depression and anxiety was already found among physicians before the COVID-19 pandemic begun. However, evidence for an increase in these over the course of the COVID-19 pandemic has been very limited so far, complicated by the fact that most previous studies depict point prevalence rates.

In the present anonymous online survey among physicians, the effects of the ongoing COVID-19 pandemic on mental wellbeing and quality of life were therefore investigated at several time points to identify risk factors for a deterioration of mental health during the COVID-19 pandemic and to derive possible preventive measures or interventions to strengthen the physicians' mental health. Following the assumptions mentioned in the introduction we therefore assume that the COVID-19 pandemic leads to an increase in anxiety and burden values over time.

Additionally, we considered whether the group of physicians who worked in COVID-19 units formed a subgroup with specific vulnerabilities or correspondingly greater negative mental health outcomes than physicians who did not work in COVID-19 units.

## 2. Materials and methods

### 2.1. Study population and recruitment methods

The survey was conducted by the Department of Addictive Behavior and Addiction Medicine at the Central Institute of Mental Health, Mannheim and the Clinic for Dermatology, Venereology and Allergology at the University Hospital Wuerzburg as an anonymous online survey in which young physicians in their first 10 years of professional life, as well as medical students from the 1st semester onwards, could participate. The survey was available online between December 1^st^, 2021, and March 31^st^, 2022. Participants were recruited with the support of the public relations departments of the participating hospitals and secretariats of the individual departments, as well as the dean's offices and student departments of the corresponding universities by recruiting participants via email.

For the survey, the software SoSci Surveys (version 2.5.00-im SoSci Survey GmbH, Munich, Germany) was used, which allows anonymous data collection without storing the IP address of the participant. Participants were informed about the content, aim and procedure of the survey before taking part in the study and had to actively give their consent to participate in the study.

The analysis presented here is a partial analysis of the total data set (*N* = 668), in which only the data of the physicians (*N* = 107) were included. The results of the students surveyed are published elsewhere (16). Moreover, in the following, not only the total sample of physicians is considered, but the subsamples “work in COVID-19 units” and “no work in COVID-19 units” (“Have you worked specifically with COVID-19 patients”—yes vs. no) are also formed and compared within the analyses.

Before the start of recruitment, positive votes were granted by the Ethics Committee II of the Mannheim Medical Faculty of the University of Heidelberg and the Ethics Committee of the University of Wuerzburg (file number MA: 2021-645; WÜ: 2021-120901). The study has been registered in the German Clinical Trials Registry (DRKS-ID: DRKS00028984).

### 2.2. Survey procedure

The self-assessment questionnaire was composed of a mixture of existing, validated and proven questionnaires and self-developed questions.

The introductory part of the questionnaire encompassed the content and objectives of the online survey as well as information on consent and data protection to ensure voluntary participation as well as anonymity and confidentiality.

The survey was broadly divided into three sections.

The first section consisted of questions about participants' sociodemographic data (age, gender, marital status, socioeconomic status, working experience and situation).

The second section of the questionnaire asked COVID-19 specific questions. It was recorded whether the subjects had worked specifically with COVID-19 patients in COVID-19 units (yes/no), how they rated the available infection protection measures and equipment in hospitals and universities (5-level from “not at all sufficient”—“completely sufficient”), the satisfaction of received appreciation from colleagues and policies (5-level from “not at all satisfied”—“completely satisfied”), the overall threat of the COVID-19 pandemic to oneself, Germany and the whole world (“low”—“medium”—“high”) and the effects on family life, social relationships and work/profession (“positive”—“negative”—“neutral”).

The final section of the survey consisted of questions about mental health before and during the COVID-19 pandemic.

Questions were also asked about pre-existing mental health conditions (yes/no) and diagnosed mental health conditions during the COVID-19 pandemic (yes/no). In addition, subjects were asked to retrospectively assess their perceived anxiety (“How would you rate your personal perceived anxiety during the pandemic?,” 3-level “none at all”—“severe”) at 7 measurement time points (spring 2020, summer 2020, fall 2020, winter 2020, spring 2021, summer 2021, fall 2021). Perceived burden (“Rate your personal stress during the pandemic,” 5-level “none”—“very high”) was also retrospectively assessed at the 7 measurement time points mentioned.

To assess symptoms of depression and anxiety among the participants, the established (α = 0.82–0.92, good validity) Hospital Anxiety and Depression Scale (HADS) (17) was administered, which contains two subscales with seven items each and a value range of 0–21, whereby higher values indicate greater depressiveness or anxiousness. Anxiety and depression were assessed on a 4-point scale (“not at all”—“most of the time”) and were rated by the subjects for the period before the outbreak of the pandemic as well as since the outbreak.

In addition, participants' quality of life was assessed using the 26-item short version of the WHO Quality of Life (WHOQOL), the WHOQOL-BREF (18), which measures the dimensions of global wellbeing, physical wellbeing, psychological wellbeing, social relationships, and environment. The questions assessing quality of life were recorded on a 5-point scale (“not at all”—“completely”) and achieved reliability values between α = 0.57 and α = 0.88.

### 2.3. Statistical analysis

Statistical calculations were performed using IBM SPSS version 27 (IBM Corporation, Armonk, New York). The 2-sided significance level was set to α = 0.05 for all tests. Frequency distributions across categories for the sociodemographic variables, the COVID-19 and working-associated questions about on-site protective measures, working in COVID-19 units, working experience, and possible psychosocial support in dealing with work, were reported as absolute numbers of cases and percentage frequencies relative to the total sample and the two subgroups formed from the total data set (participants with COVID-19 unit assignments and participants without COVID-19 unit assignments).

The significance of differences in subjective anxiety and burden over time from spring 2020 to fall 2021 between the seven measurement time points was tested using the Friedman non-parametric test. Means and standard deviations were reported for the HADS and WHOQOL-BREF for the total group and the two subgroups. Comparisons of the sum scores of the HADS before and after the onset of the COVID-19 pandemic were performed using paired-sample *t*-tests for both the total group and subsamples. Differences in mean quality of life scores (WHOQOL-BREF) between subsamples (participants working in COVID-19 units vs. participants not working in COVID-19 units) were tested for significance using independent sample *t*-tests.

A multi-factorial ANOVA was calculated to analyze the influence of age, gender, presence of a previous mental illness before the pandemic, availability of infection protection measures, mean subjective anxiety, mean burden, change in HADS sum score from before to after the outbreak of the pandemic, and professional experience on the current quality of life of the physicians.

## 3. Results

### 3.1. Sample description

The data of *N* = 107 physicians aged 23–42 years (*M* = 30.07, SD = 4.22) were included in the analyses. *N* = 66 (61.7%) of participants were male, *N* = 41 (38.3%) female. The physicians indicated work experience of 1–2 years (*N* = 43, 39.8%), 3–7 years (*N* = 47, 43.5%) and 8–11 years (*N* = 17, 15.7%). Participating physicians worked in many different areas: *N* = 3 general medicine, *N* = 8 anesthesiology, *N* = 8 surgery, *N* = 5 gynecology, *N* = 2 otorhinolaryngologist, *N* = 3 dematology, *N* = 1 endocrinology, *N* = 15 internal medicine, *N* = 10 pediatrics, *N* = 6 child and adolescent psychiatry, *N* = 1 laboratory medicine, *N* = 1 infectious disease epidemiology, *N* = 2 oral and maxillofacial surgery, *N* = 2 neurosurgery, *N* = 3 neurology, *N* = 1 public health, *N* = 4 psychiatry and psychotherapy, *N* = 2 radiation therapy, *N* = 3 transfusion medicine, *N* = 1 urology, *N* = 2 dental medicine.

Analyses were performed for the total sample of all participants as well as for the subgroup of physicians working in COVID-19 units at the time of the survey (*N* = 56, 52.3%) and the subgroup of physicians not working in COVID-19 units (*N* = 51, 47.7%).

An overview of the sociodemographic variables (gender, marital status, professional experience, and socioeconomic status, as well as mean age) for both the total sample and the subsamples is given in Table 1.

**Table 1:** Sociodemographics and COVID-19 situation.

	**Total sample**		**Work in COVID-19 units**		**No work in COVID-19 units**	
	***N***	**%**	***N***	**%**	***N***	**%**
	107	100	56	52.3	51	47.7
**Gender**						
Female	66	61.7	31	55.4	35	68.6
Male	41	38.3	25	44.6	16	31.4
**Family status**						
Married	30	28.0	21^*^	37.5	9^*^	17.6
Single/single living	32	29.9	12^*^	21.4	20^*^	39.2
Liaised/engaged	7	6.5	6	10.7	1	6.5
Living with partner	33	30.8	14	25.0	19	30.8
Living separately	1	0.9	1	1.8	0	0
Divorced	1	0.9	0	0	1	2.0
Others	3	2.8	2	3.6	1	2.0
**Socioeconomic status**						
Low	0	0	0	0	0	0
Insufficient	0	0	0	0	0	0
Medium	17	15.9	5	8.9	12	23.5
Sufficient	49	45.8	29	51.8	20	39.2
High	41	38.3	22	39.3	19	37.3
**Work experience**						
1–2 years	43	39.8	17	30.4	26	51.0
3–7 years	47	43.5	26	46.4	21	41.2
8–11 years	17	15.7	13	23.2	4	7.8
Age in years (*M*, SD)	30.07 (4.22)		30.87^*^ (4.02)		29.18^*^ (4.29)	

Persons with missing data were excluded.

Socioeconomic status corresponds to a subjective assessment without a objective basis.

Protection equipment was queried on a 5-point Likert scale from 1 = not at all sufficient to 5 = fully sufficient.

N, sample size; M, mean; SD, standard deviation;

* Values differ significantly between “work in COVID-19 units” and “no work in COVID-19 units” subgroups, p < 0.05.

### 3.2. Subjectively perceived anxiety

**Figure 1:**
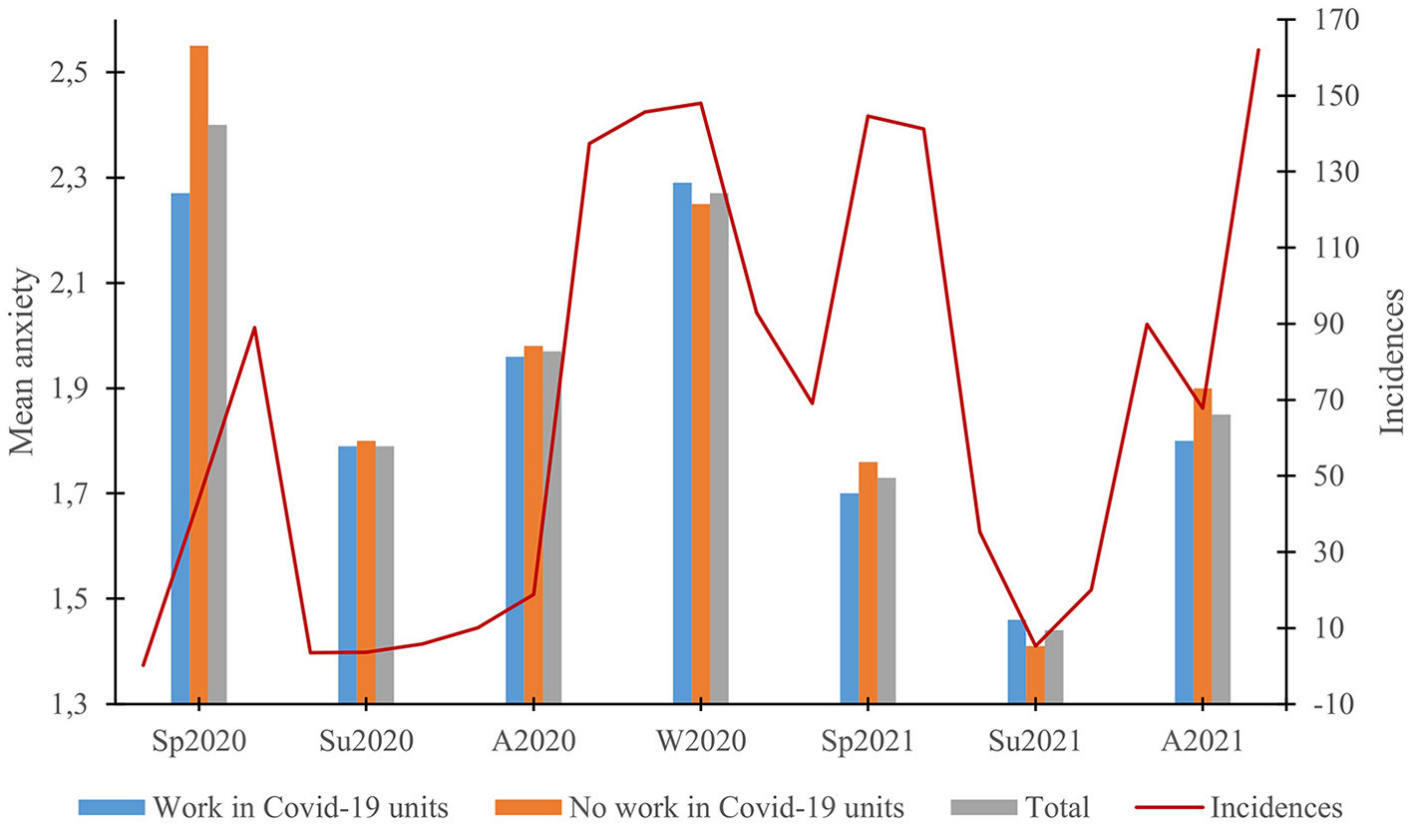
Subjective anxiety over time. Presentation of mean anxiety scores at the different measurement time points for the total sample and the “work in COVID-19 units” and “no work in COVID-19 units” subsamples in relation to the nationwide COVID-19 7-day incidence rate over time at the beginning of each month (20/03: 0,2; 20/04: 44; 20/05: 89; 20/06: 3,5; 20/07: 3,6; 20/08: 5,8; 20/09: 10,1; 20/10: 18,8; 20/11: 137,4; 20/12: 145,7; 21/01: 148; 21/02: 92,9; 21/03: 69,1; 21/04: 144,6; 21/05: 141,2; 21/06: 35,3; 21/07: 5,3; 21/08: 20; 21/09: 89,9; 21/10: 67,8; 21/11: 162) Source: Robert Koch Institut (RKI): COVID-19 trends in Germany in an overview: https://www.rki.de/DE/Content/InfAZ/N/Neuartiges_Coronavirus/Situationsberichte/COVID-19-Trends/COVID-19-Trends.html?__blob$=$publicationFile#/home; Access: 02.05.2022.

Subjective anxiety of physicians differed significantly between the seven measurement time points both in the total sample [Friedman test: Chi^2^(6) = 197.05, *p* < 0.001, *n* = 107] and in the subsample “working in COVID-19 units” [Chi^2^(6) = 95.24, *p* < 0.001, *n* = 56] and the subsample “not working in COVID-19 units” [Chi^2^(6) = 103.78, *p* < 0.001, *n* = 51]. The curve of the anxiety scores followed a wave-like progression similar to that of the COVID-19 incidence, with higher scores in fall, winter, and spring months and lower scores in summer months (see Figure 1), with mean anxiety score wave crests (in spring-autumn and winter months) or wave troughs (summer months) resulting higher in 2020 than in 2021. The results of the post-hoc Dunn-Bonferroni tests, which test each measurement time point against the other, can be found in the online Supplementary material.

### 3.3. Subjectively perceived burden

**Figure 2:**
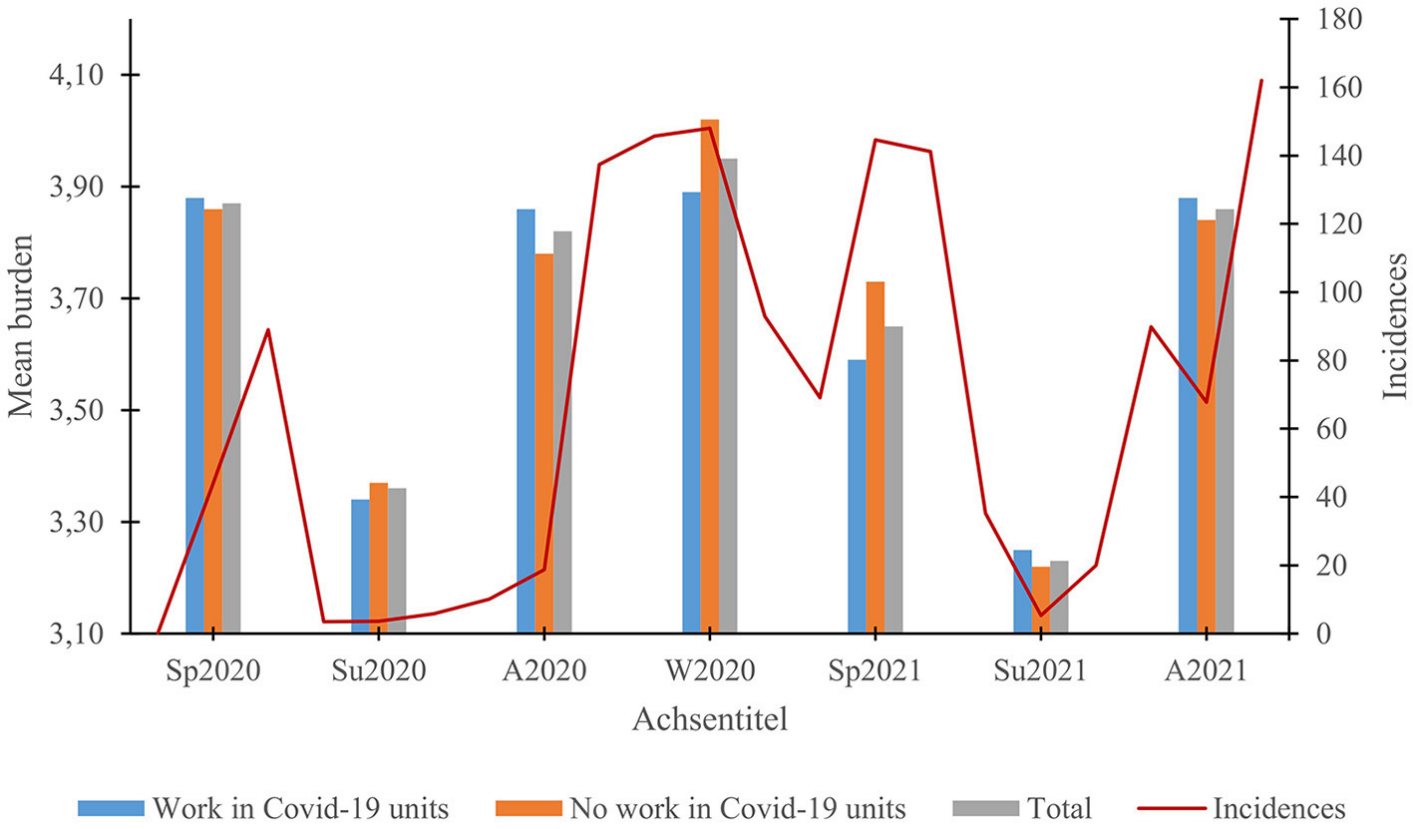
Subjective burden over time. Presentation of mean burden scores at the different measurement time points for the total sample and the “work in COVID-19 units” and “no work in COVID-19 units” subsamples in relation to the nationwide COVID-19 7-day incidence rate over time at the beginning of each month (for exact scores see Figure 1).

Significant differences between all seven measurement time points were also evident with respect to the subjective burden of physicians during the course of the COVID-19 pandemic in both the overall sample [Friedman test: Chi^2^(6) = 106. 33, *p* < 0.001, *n* = 107] as well as in the “working in COVID-19 units” subsample [Chi^2^(6) = 65.58, *p* < 0.001, *n* = 56] and the “not working in COVID-19 units” subsample [Chi^2^(6) = 43.93, *p* < 0.001, *n* = 51] (Figure 2). Subjective burden in the summer months of 2020 and 2021 was significantly lower than in the spring, fall, and winter months of the sampled period. In comparison to periods in 2020, there were no lower mean scores in 2021. A detailed account of the post-hoc Dunn-Bonferroni tests can be found in the online Supplementary material.

### 3.4. Change in depression scores

**Figure 3:**
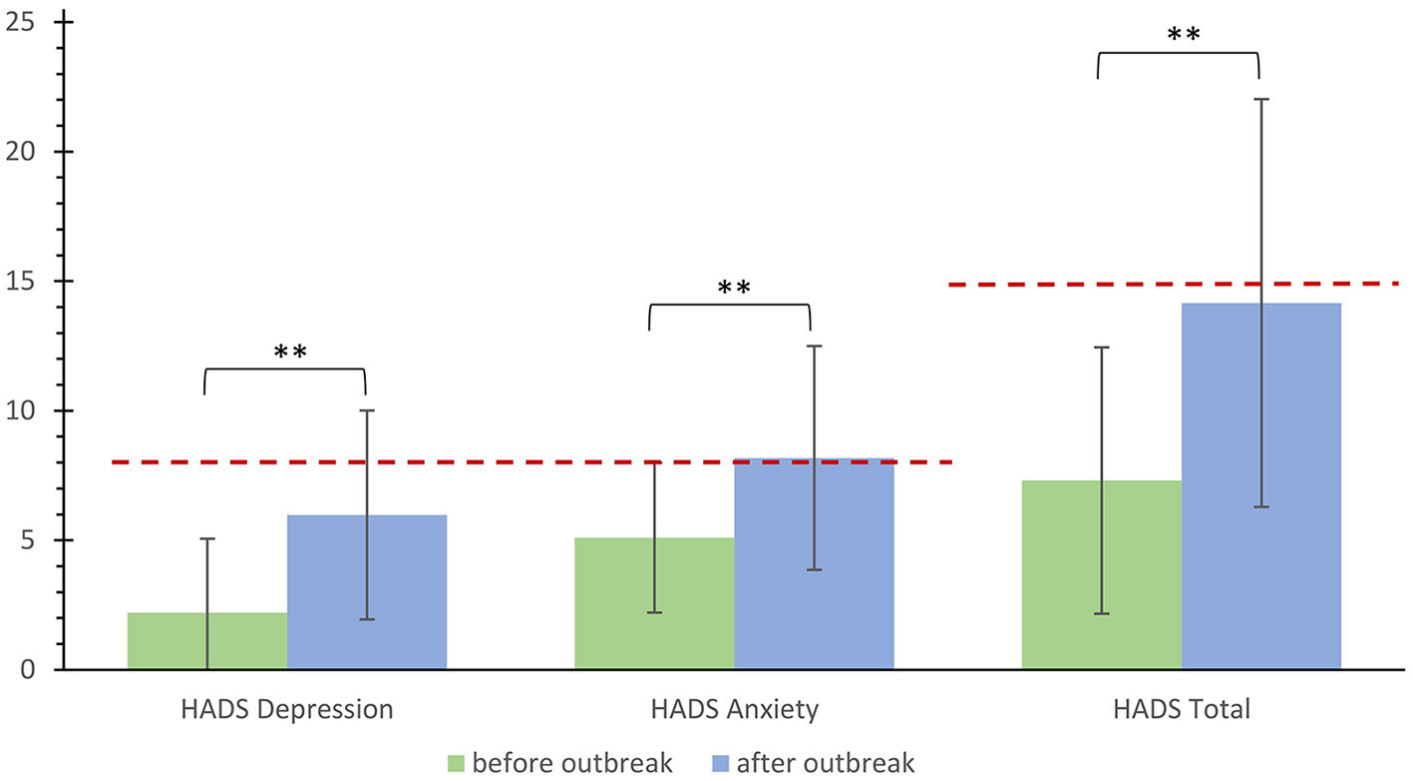
Change in HADS sum scores. Change in HADS sum scores for the HADS total scale and the anxiety and depression subscales before the outbreak of the pandemic compared to after the outbreak of the pandemic. The dashed line corresponds to the cut-offs for a clinically abnormal score. **Significant differences, *p* < 0.05.

Mean depression scores after the onset (ao) of the COVID-19 pandemic significantly increased compared to mean depression scores before the onset (bo) of the pandemic for both the total scale of the HADS [bo: *M* = 7.31, SD = 5.14; ao: *M* = 14.16, SD = 7. 83, *t*_(106)_ = −10.67, *p* < 0.001], as well as the depression subscale [bo: *M* = 2.21, SD = 2.85; ao: *M* = 5.98, SD = 4.03; *t*_(106)_ = −10.62, *p* < 0.001] and the anxiety subscale [bo: *M* = 5.10, SD = 2.89; ao: *M* = 8.18, SD = 4.32, *t*_(106)_ = −9.36, *p* < 0.001]. It seems particularly relevant that before the outbreak of the COVID-19 pandemic only 9.3% of all physicians exceeded the cut-off of 15 to a clinically questionable value, compared to after the outbreak where it reached 42.1%. A similar picture was seen in the subscales with the cut-off of 8 points to a clinically conspicuous value (depression: bo: 3.7% ≥ 8, ao: 23.4% ≥ 8; anxiety: bo: 21.5% ≥ 8, ao: 54.2% ≥ 8, see Figure 3).

Physicians working in COVID-19 units (wC) showed a significantly smaller increase in depression scores after the onset of the COVID-19 pandemic compared to the time before than physicians not working in COVID-19 units (nC). These significant differences were evident for both the total scale of the HADS [wC: *M* = 2.88, SD = 2.83; nC: *M* = 4.76, SD = 4.24, *t*_(85.97)_ = −2.687, *p* = 0.009] as well as for the subscales anxiety [wC: *M* = 2.18, SD = 2.79; nC: *M* = 4.06, SD = 3.74, *t*_(91.98)_ = −2.923, *p* = 0.004] and depression [wC: *M* = 5.05, SD = 5.13; nC: *M* = 8.82, SD = 7.55, *t*_(86.93)_ = −2.993, *p* = 0.004].

### 3.5. Subjective quality of life after 2 years of pandemic

Table 2 provides an overview of participants' subjective quality of life in the different domains of the WHOQOL-BREF for the total sample and the subsamples “working in COVID-19 units” and “not working in COVID-19 units” at the interview time point 2 years after the COVID-19 pandemic begun.

**Table 2:** Participants' subjective quality of life after 2 years of pandemic according to WHOQOL-BREF.

	**Total sample**		**Work in COVID-19 units**		**No work in COVID-19 units**	
	***M***	**SD**	***M***	**SD**	***M***	**SD**
WHO Domain1: Global	68.46	21.61	66.96	20.56	70.10	22.79
WHO Domain2: Physical	76.20	17.22	77.61	16.01	74.65	18.49
WHO Domain3: Psychological	67.33	18.59	68.90	17.55	65.60	19.70
WHO Domain4: Social relationships	66.12	21.61	70.39^**^	17.69	61.44^**^	24.55
WHO Domain5: Environmental	75.61	14.84	76.73	13.15	74.39	16.54

M, mean; SD, standard deviation;

** Significant differences, p < 0.001.

The “working in COVID-19 units” subsample had significantly higher scores for the quality of life domain related to social relationships than the “not working in COVID-19 units” subsample [*t*_(90.14)_ = −2.145, *p* = 0.035, |*d*| = 0.421]. The subsamples did not differ in the domains of quality of life globally [*t*_(105)_ = −0.748, *p* = 0.456], physically [*t*_(105)_ = 0.889, *p* = 0.376], psychologically [*t*_(105)_ = 0.915, *p* = 362], and in relation to the environment [*t*_(105)_ = 0.815, *p* = 0.417].

### 3.6. Factors influencing the quality of life

The multifactorial ANOVA showed that the presence of a previous mental illness [*F*_(1,97)_ = 16.520, *p* < 0.001, $\eta_{p}^{2}$ = 0.146], the difference in depression scores before and after the outbreak of the pandemic [*F*_(1,97)_ = 8.703, *p* = 0.004, $\eta_{p}^{2}$ = 0.082], mean anxiety [*F*_(1,97)_ = 4.009, *p* = 0.048, $\eta_{p}^{2}$ = 0.040], and work experience [*F*_(1,97)_ = 3.679, *p* = 0.029, $\eta_{p}^{2}$ = 0.071] had a significant association with or significant main effect on quality of life related to social relationships.

The presence of a previous mental illness [*B* = −22.173, *t*_(106)_ = −4.064, *p* < 0.001], a greater difference in depression scores [*B* = −0.924, *t*_(106)_ = −2.950, *p* = 0.004], higher anxiety scores [*B* = 8. 113, *t*_(106)_ = 2.002, *p* = 0.048] and lower work experience [*B* = −16.688, *t*_(105)_ = −1.965, *p* = 0.052] lead to lower scores in quality of life related to social relationships.

The overall model was significant, explaining 35.0% of the variance in quality of life related to social relationships [*F*_(9, 97)_ = 7.332, *p* < 0.001, adjusted *R*^2^ = 0.350, *n* = 107, $\eta_{p}^{2}$ = 0.405].

The variables gender (*p* = 0.085), presence of infection control measures (*p* = 0.318), mean burden (*p* = 0.109), and age (*p* = 0.913) showed no significant association with quality of life in relation to social relationships.

## 4. Discussion

Similar to the wave-like COVID-19 incidence rates in Germany, the subjective anxiety and personal burden of physicians showed higher values in the autumn, winter and spring months. The anxiety values in 2021 were significantly lower than in 2020, whereas the burden values did not differ between 2020 and 2021.

The comparability of our results on the extent of anxiety and subjective burden with previous studies appears to be hampered by the fact that these were mostly cross-sectional surveys reporting only point prevalence rates [at the beginning of the pandemic: 11% (6), during the pandemic: 17% (14), 20.5% (9), 21.7% (19)]. In comparison, previous studies showed higher values during the COVID-19 pandemic than at the beginning, but they did not report comparative values within a sample. Our study, on the other hand, retrospectively collected follow-up values at 3-month intervals, thus intra-individual comparisons could depict the development of anxiety over time. The reason for the overall lower anxiety scores in 2021 than in 2020 could be explained by a possible habituation effect to the pandemic living conditions. As the pandemic progresses, the COVID-19 virus and the associated symptoms and dangers are no longer unknown to physicians. Vaccines and initial treatment options become available, so the COVID-19-associated fear and uncertainty reduce overall. The burden of the COVID-19 pandemic, on the other hand, did not decrease among physicians between 2020 and 2021. Consistent with the incidence rates, there were also a large number of COVID-19 cases in hospitals in 2021, which was associated with an increased burden for physicians. At the beginning of the COVID-19 pandemic there was a high level of confidence that the pandemic could be managed quickly, and governmental relief such as financial rescue packages were available. At the same time the occupancy pressure in the wards increased as the pandemic continued, along with an increased number of staff absences (20) and a loss of faith in the quick end of the COVID-19 pandemic (21). Furthermore, in 2021 there were still increased hygiene requirements in hospitals, which made the work of physicians more difficult and heightened their burden.

Furthermore, physicians showed significantly higher depression scores after the outbreak of the COVID-19 pandemic than before the outbreak, with those working in COVID-19 units showing a lower increase than those who did not work directly in COVID-19 units. The physicians working in COVID-19 units also showed higher quality of life scores in relation to social relationships than the physicians who did not work in these units, with the presence of a previous mental illness, a greater difference in depression scores, higher anxiety scores and less work experience leading to lower quality of life scores.

Compared to previous studies, the physicians in our sample retrospectively reported significantly lower mean depression scores at the beginning of the pandemic (13). The above mentioned significantly higher depression scores after the outbreak of the pandemic are consistent with the pooled prevalence rates of meta-analyses (9, 19).

The significantly lower increase in depression scores after the onset of the COVID-19 pandemic and the higher scores for quality of life in relation to social relationships among physicians working in COVID-19 units compared to physicians not working in COVID-19 units contradicts results of previous studies that did not find differences between these two groups of physicians in terms of anxiety, depression or burden (8).

The differences we found could be due to the fact that doctors working in COVID-19 units adapted more quickly to the challenges associated with work-related implications of the pandemic due to the increased exposure and were able to possibly transfer this into their private lives. It is possible, that social relationships are part of the coping mechanisms for the increased burden of working in COVID-19 units. These may have a positive impact on quality of life, while at the same time social relationships and quality of life may suffer in more depressed individuals (in this case physicians not working in COVID-19 units) (22).

Following the differences in German and Australian physicians with respect to coping mechanisms (9), our results may suggest that individuals working in COVID-19 units, like Australian physicians, are more likely to use more emotional coping strategies (such as a focus on social relationships) and consequently experience less stress, burden, and depression-associated symptoms.

Consistent with these findings, physicians who worked in COVID-19 units and showed higher social relationship quality of life scores were significantly more likely to be married than individuals who did not work in COVID-19 units. These physicians, in turn, showed lower quality of life scores related to social relationships and were significantly more likely to report “single/single living” as their relationship status than physicians in COVID-19 units. Being married and the associated social support could be protective factors in this context and could contribute to the higher quality of life in terms of social relationships.

Furthermore, physicians working in COVID-19 units had much earlier access to protective measures and equipment as well as immunization (23). The shared “fight” against the COVID-19 pandemic in a team and the mutual social support probably also had a positive effect on symptoms of depression and quality of life.

Another factor that could have lead to a greater increase in depression scores among physicians not working in COVID-19 units is, that these physicians were often no longer able to fully perform their actual professional activities due to restrictions (e.g., by canceling elective operations). This could have led to frustration due to different public recognition for the different groups of healthcare workers, while at the same time being confronted with challenges such as an increased need for testing and protective measures, even in normal wards and finally being associated with a negative impact on physician emotional wellbeing (24).

Several limitations should be noted when interpreting the results. First, the survey is an anonymous online survey. With this method, by its very nature, the quality of answers and the population cannot be objectively described. Respondents may be preselected and may therefore not be a representative sample. At the same time, the online survey method is an economical means of collecting larger samples and reaching individuals who might not participate in a face-to-face study due to time constraints, for example.

A further limitation arises from the fact that the retrospective survey of mental status, on the one hand, did not specify exact points in time but only time periods and, on the other hand, with such a long period of time, there could be errors in the exact recollection of the physicians. Third, the results are analyses of a rather small sample of physicians, *N* = 107, which may limit the representativeness of the results. Since we excluded incomplete responses, this might have further reduced the number of analyzed responses. Nevertheless, it should be emphasized that our relatively small study sample meets the characteristics of a representative sample of physicians. For example, many different specialties are covered in our study such as descriped in the sample description section. In addition the distribution of age is similar to those in the general population, as we surveyed physicians in the first 10 years of their careers and had an average age of 30 years in the sample (see sample description), while according to statistics, entry-level physicians in Germany are 26.1 years old (25). To the best of our knowledge, this was the first study in Germany to capture early physician burden in the Corona pandemic, complicated by the timely and emotionally constrained resources and thus limited willingness of physicians to participate in studies. Consequently, the results presented should be seen as a pilot study with promising results that should being confirmed in a survey with a larger sample among others to confirm representiveness. A further limitation results from the overrepresentation of the female gender, with 61.7% female participants in our sample compared to the reference collective of all physicians in Germany [49.9% female (26)]. Despite these limitations, this study represents one of the first surveys regarding the impact of the COVID-19 pandemic on physicians' mental health and an assessment of the progression of distress during the pandemic.

## 5. Conclusions

Despite the limitations mentioned above, the results of the present study suggest that the ongoing COVID-19 pandemic leads to a sustained increase in the psychological burden of physicians and consequently, has a negative impact on their quality of life. In order to counteract this increased psychological stress and to prevent the development of psychological sequelae such as chronic depression or burnout with long-term sick leave and high costs for the health care system, prevention and support such as supervision or Balint groups should be established promptly, especially for younger doctors with less professional experience.

## Data availability statement

The raw data supporting the conclusions of this article will be made available by the authors, without undue reservation.

## Ethics statement

The studies involving human participants were reviewed and approved by Ethics Committee II of the Mannheim Medical Faculty of the University of Heidelberg and the Ethics Committee of the University of Wuerzburg. The patients/participants provided their written informed consent to participate in this study.

## Author contributions

LW, MH, AS, AK, FK, and SK conceived of the study. LW, MH, NC, AS, and AK initiated the study design and implementation of the study. Statistics were carried out by LW, MH, AS, and AK. The manuscript was prepared by LW and MH as lead authors as well as FK, AS, and AK. All authors contributed to refinement of the study protocol and approved the final manuscript.
