# Supplementary material for: The impact of COVID-19 pandemic on mental burden and quality of life in physicians: Results of an online survey
Source: Front Psychiatry. 2023 Apr 13;14:1068715. doi: 10.3389/fpsyt.2023.1068715 (PMC10133485; doi:10.3389/fpsyt.2023.1068715)
Supplement: Supplementary file 1 [file Table_1.DOCX]

| *Table 3: Scores of subjective anxiety of the subjects at the various time points* | | | | | | | |
| --- | --- | --- | --- | --- | --- | --- | --- |
|  |  | Total sample | | Work in Covid-19 units | | No work in Covid-19 units | |
|  |  | M | SD | M | SD | M | SD |
| Spring 2020 | | 2.40 | .71 | 2.27 | .75 | 2.55 | .64 |
| Summer 2020 | | 1.79 | .61 | 1.79 | .59 | 1.80 | .63 |
| Autumn 2020 | | 1.97 | .71 | 1.96 | .69 | 1.98 | .74 |
| Winter 2020 | | 2.27 | .73 | 2.29 | .76 | 2.25 | .72 |
| Spring 2021 | | 1.73 | .73 | 1.70 | .71 | 1.76 | .76 |
| Summer 2021 | | 1.44 | .57 | 1.46 | .57 | 1.41 | .57 |
| Autumn 2021 | | 1.85 | .71 | 1.80 | .72 | 1.90 | .70 |

M: mean; SD: standard deviation

| *Table 4: Scores of subjective burden of the subjects at the various time points* | | | | | | | |
| --- | --- | --- | --- | --- | --- | --- | --- |
|  |  | Total sample | | Work in Covid-19 units | | No work in Covid-19 units | |
|  |  | M | SD | M | SD | M | SD |
| Spring 2020 | | 3.87 | .92 | 3.87 | .83 | 3.86 | 1.02 |
| Summer 2020 | | 3.36 | .84 | 3.34 | .75 | 3.37 | .94 |
| Autumn 2020 | | 3.82 | .80 | 3.86 | .77 | 3.78 | .83 |
| Winter 2020 | | 3.95 | .84 | 3.89 | .93 | 4.02 | .74 |
| Spring 2021 | | 3.65 | .83 | 3.59 | .87 | 3.73 | .78 |
| Summer 2021 | | 3.23 | .82 | 3.25 | .75 | 3.22 | .90 |
| Autumn 2021 | | 3.86 | .87 | 3.88 | .85 | 3.84 | .90 |

M: mean; SD: standard deviation

| *Table 5: Factors influencing quality of life* | | | | |
| --- | --- | --- | --- | --- |
|  | df | F | Sig. | η_p_^2^ |
| Gender | 1 | 3.029 | .085 | .030 |
| Previous mental illness | 1 | 16.520 | .000* | .146 |
| Infection control measures | 1 | 1.007 | .318 | .010 |
| Differences in HADS | 1 | 8.703 | .004* | .082 |
| Mean Anxiety | 1 | 4.009 | .048* | .040 |
| Mean Burden | 1 | 2.622 | .109 | .026 |
| Age | 1 | .012 | .913 | .000 |
| Work experience | 2 | 3.679 | .029* | .071 |
| R^2^ = .405 (adjusted R^2^ = .350); df: degrees of freedom; F: F-value; η_p_^2^ : partial Eta^2;^ * significant factor, p<.05. | | | | |

| *Table 6: Parameter estimation of factors influencing quality of life* | | | | |
| --- | --- | --- | --- | --- |
|  | B | T | Sig. | η_p_^2^ |
| Gender (= female) | 6.655 | 1.740 | .085 | .030 |
| Gender (= male) | 0 | . | . | . |
| Previous mental illness (= yes) | -22.173 | -4.064 | .000* | .146 |
| Previous mental illness (= no) | 0 | . | . | . |
| Infection control measures | 2.135 | 1.003 | .318 | .010 |
| Differences in HADS | -.924 | -2.950 | .004* | .082 |
| Mean Anxiety | 8.113 | 2.002 | .048* | .040 |
| Mean Burden | -6.447 | -1.619 | .109 | .026 |
| Age | .075 | .110 | .913 | .000 |
| Work experience (= 1-2 years) | -16.688 | -1.965 | .052 | .038 |
| Work experience (= 3-7 years) | -5.124 | -.729 | .468 | .005 |
| Work experience (= 8-11 years) | 0 | . | . | . |
| B: regression coefficient; T: T-value; η_p_^2^ : partial Eta^2^* significant factor, p<.05. | | | | |
