# Supplementary material for: The impact of COVID-19 pandemic on mental burden and quality of life in physicians: Results of an online survey
Source: Front Psychiatry. 2023 Apr 13;14:1068715. doi: 10.3389/fpsyt.2023.1068715 (PMC10133485; doi:10.3389/fpsyt.2023.1068715)
Supplement: Supplementary file 2 [file Table_2.docx]

| *Pairwise comparisons, post-hoc Dunn-Bonferroni tests; subjective anxiety, total* | | | | | |
| --- | --- | --- | --- | --- | --- |
| Sample 1-Sample 2 | Test Statistics | Standard Error | Standard Test Statistics | Sig. | Adap. Sig.^a^ |
| Anxiety_Su_2021-Anxiety_Su_2020 | ,738 | ,295 | 2,500 | ,012 | ,261 |
| Anxiety_Su_2021-Anxiety_Sp_2021 | ,930 | ,295 | 3,149 | ,002 | ,034 |
| Anxiety_Su_2021-Anxiety_A_2020 | -1,187 | ,295 | -4,019 | ,000 | ,001 |
| Anxiety_Su_2021-Anxiety_A_2021 | 1,547 | ,295 | 5,237 | ,000 | ,000 |
| Anxiety_Su_2021- Anxiety _Sp_2020 | 2,421 | ,295 | 8,196 | ,000 | ,000 |
| Anxiety_Su_2021-Anxiety_W_2020 | 2,729 | ,295 | 9,240 | ,000 | ,000 |
| Anxiety_Su_2020-Anxiety_Sp_2021 | ,192 | ,295 | ,649 | ,517 | 1,000 |
| Anxiety_Su_2020-Anxiety_A_2020 | -,449 | ,295 | -1,519 | ,129 | 1,000 |
| Anxiety_Su_2020-Anxiety_A_2021 | ,808 | ,295 | 2,737 | ,006 | ,130 |
| Anxiety_Su_2020-Anxiety_Sp_2020 | 1,682 | ,295 | 5,696 | ,000 | ,000 |
| Anxiety_Su_2020-Anxiety_W_2020 | 1,991 | ,295 | 6,740 | ,000 | ,000 |
| Anxiety_Sp_2021-Anxiety_A_2020 | -,257 | ,295 | -,870 | ,384 | 1,000 |
| Anxiety_Sp_2021-Anxiety_A_2021 | -,617 | ,295 | -2,088 | ,037 | ,772 |
| Anxiety_Sp_2021-Anxiety_Sp_2020 | -1,491 | ,295 | -5,047 | ,000 | ,000 |
| Anxiety_Sp_2021-Anxiety_W_2020 | 1,799 | ,295 | 6,091 | ,000 | ,000 |
| Anxiety_A_2020-Anxiety_A_2021 | ,360 | ,295 | 1,218 | ,223 | 1,000 |
| Anxiety_A_2020-Anxiety_Sp_2020 | 1,234 | ,295 | 4,177 | ,000 | ,001 |
| Anxiety_A_2020-Anxiety_W_2020 | 1,542 | ,295 | 5,221 | ,000 | ,000 |
| Anxiety_A_2021-Anxiety_Sp_2020 | -,874 | ,295 | -2,959 | ,003 | ,065 |
| Anxiety_A_2021-Anxiety_W_2020 | 1,182 | ,295 | 4,003 | ,000 | ,001 |
| Anxiety_Sp_2020-Anxiety_W_2020 | ,308 | ,295 | 1,044 | ,296 | 1,000 |

| Each row tests the null hypothesis that the distributions in sample 1 and sample 2 are the same. |
| --- |
| Asymptotic significances (two-sided tests) are shown.   1. The significance level is .050. |

| *Pairwise comparisons, post-hoc Dunn-Bonferroni tests; subjective anxiety, work in COVID-19 units* | | | | | |
| --- | --- | --- | --- | --- | --- |
| Sample 1-Sample 2 | Test Statistics | Standard Error | Standard Test Statistics | Sig. | Adap. Sig.^a^ |
| Anxiety_Su_2021-Anxiety_Su_2020 | ,652 | ,408 | 1,597 | ,110 | 1,000 |
| Anxiety_Su_2021-Anxiety_Sp_2021 | ,804 | ,408 | 1,968 | ,049 | 1,000 |
| Anxiety_Su_2021-Anxiety_A_2020 | -1,009 | ,408 | -2,471 | ,013 | ,283 |
| Anxiety_Su_2021-Anxiety_A_2021 | 1,438 | ,408 | 3,521 | ,000 | ,009 |
| Anxiety_Su_2021- Anxiety _Sp_2020 | 2,357 | ,408 | 5,774 | ,000 | ,000 |
| Anxiety_Su_2021-Anxiety_W_2020 | 2,366 | ,408 | 5,796 | ,000 | ,000 |
| Anxiety_Su_2020-Anxiety_Sp_2021 | ,152 | ,408 | ,372 | ,710 | 1,000 |
| Anxiety_Su_2020-Anxiety_A_2020 | -,357 | ,408 | -,875 | ,382 | 1,000 |
| Anxiety_Su_2020-Anxiety_A_2021 | ,786 | ,408 | 1,925 | ,054 | 1,000 |
| Anxiety_Su_2020-Anxiety_Sp_2020 | 1,705 | ,408 | 4,177 | ,000 | ,001 |
| Anxiety_Su_2020-Anxiety_W_2020 | 1,714 | ,408 | 4,199 | ,000 | ,001 |
| Anxiety_Sp_2021-Anxiety_A_2020 | -,205 | ,408 | -,503 | ,615 | 1,000 |
| Anxiety_Sp_2021-Anxiety_A_2021 | -,634 | ,408 | -1,553 | ,120 | 1,000 |
| Anxiety_Sp_2021-Anxiety_Sp_2020 | 1,554 | ,408 | 3,805 | ,000 | ,003 |
| Anxiety_Sp_2021-Anxiety_W_2020 | -1,562 | ,408 | -3,827 | ,000 | ,003 |
| Anxiety_A_2020-Anxiety_A_2021 | ,429 | ,408 | 1,050 | ,294 | 1,000 |
| Anxiety_A_2020-Anxiety_Sp_2020 | 1,348 | ,408 | 3,302 | ,001 | ,020 |
| Anxiety_A_2020-Anxiety_W_2020 | 1,357 | ,408 | 3,324 | ,001 | ,019 |
| Anxiety_A_2021-Anxiety_Sp_2020 | ,920 | ,408 | 2,253 | ,024 | ,510 |
| Anxiety_A_2021-Anxiety_W_2020 | -,929 | ,408 | -2,275 | ,023 | ,482 |
| Anxiety_Sp_2020-Anxiety_W_2020 | -,009 | ,408 | -,022 | ,983 | 1,000 |

| *Pairwise comparisons, post-hoc Dunn-Bonferroni tests; subjective anxiety, no work in COVID-19 units* | | | | | |
| --- | --- | --- | --- | --- | --- |
| Sample 1-Sample 2 | Test Statistics | Standard Error | Standard Test Statistics | Sig. | Adap. Sig.^a^ |
| Anxiety_Su_2021-Anxiety_Su_2020 | ,833 | ,428 | 1,948 | ,051 | 1,000 |
| Anxiety_Su_2021-Anxiety_Sp_2021 | 1,069 | ,428 | 2,498 | ,012 | ,262 |
| Anxiety_Su_2021-Anxiety_A_2020 | -1,382 | ,428 | -3,231 | ,001 | ,026 |
| Anxiety_Su_2021-Anxiety_A_2021 | 1,667 | ,428 | 3,896 | ,000 | ,002 |
| Anxiety_Su_2021- Anxiety _Sp_2020 | 2,480 | ,428 | 5,798 | ,000 | ,000 |
| Anxiety_Su_2021-Anxiety_W_2020 | 3,137 | ,428 | 7,334 | ,000 | ,000 |
| Anxiety_Su_2020-Anxiety_Sp_2021 | ,235 | ,428 | ,550 | ,582 | 1,000 |
| Anxiety_Su_2020-Anxiety_A_2020 | -,549 | ,428 | -1,283 | ,199 | 1,000 |
| Anxiety_Su_2020-Anxiety_A_2021 | ,833 | ,428 | 1,948 | ,051 | 1,000 |
| Anxiety_Su_2020-Anxiety_Sp_2020 | 1,647 | ,428 | 3,850 | ,000 | ,002 |
| Anxiety_Su_2020-Anxiety_W_2020 | 2,304 | ,428 | 5,386 | ,000 | ,000 |
| Anxiety_Sp_2021-Anxiety_A_2020 | -,314 | ,428 | -,733 | ,463 | 1,000 |
| Anxiety_Sp_2021-Anxiety_A_2021 | -,598 | ,428 | -1,398 | ,162 | 1,000 |
| Anxiety_Sp_2021-Anxiety_Sp_2020 | -1,412 | ,428 | -3,300 | ,001 | ,020 |
| Anxiety_Sp_2021-Anxiety_W_2020 | 2,069 | ,428 | 4,836 | ,000 | ,000 |
| Anxiety_A_2020-Anxiety_A_2021 | ,284 | ,428 | ,665 | ,506 | 1,000 |
| Anxiety_A_2020-Anxiety_Sp_2020 | 1,098 | ,428 | 2,567 | ,010 | ,216 |
| Anxiety_A_2020-Anxiety_W_2020 | 1,755 | ,428 | 4,102 | ,000 | ,001 |
| Anxiety_A_2021-Anxiety_Sp_2020 | -,814 | ,428 | -1,902 | ,057 | 1,000 |
| Anxiety_A_2021-Anxiety_W_2020 | 1,471 | ,428 | 3,438 | ,001 | ,012 |
| Anxiety_Sp_2020-Anxiety_W_2020 | ,657 | ,428 | 1,535 | ,125 | 1,000 |
