# Supplementary material for: The impact of COVID-19 pandemic on mental burden and quality of life in physicians: Results of an online survey
Source: Front Psychiatry. 2023 Apr 13;14:1068715. doi: 10.3389/fpsyt.2023.1068715 (PMC10133485; doi:10.3389/fpsyt.2023.1068715)
Supplement: Supplementary file 3 [file Table_3.docx]

| \| *Pairwise comparisons, post-hoc Dunn-Bonferroni tests; subjective burden; total sample* \| \| \| \| \| \| \| --- \| --- \| --- \| --- \| --- \| --- \| \| Sample 1-Sample 2 \| Test Statistics \| Standard Error \| Standard Test Statistics \| Sig. \| Adap. Sig.^a^ \| \| Burden_Su_2020-Burden_Su_2021 \| ,150 \| ,295 \| ,506 \| ,613 \| 1,000 \| \| Burden_Su_2020-Burden_Sp_2020 \| ,977 \| ,295 \| 3,307 \| ,001 \| ,020 \| \| Burden_Su_2020-Burden_A_2020 \| 1,444 \| ,295 \| 4,889 \| ,000 \| ,000 \| \| Burden_Su_2020-Burden_Sp_2021 \| -1,505 \| ,295 \| -5,095 \| ,000 \| ,000 \| \| Burden_Su_2020-Burden_A_2021 \| 1,533 \| ,295 \| 5,190 \| ,000 \| ,000 \| \| Burden_Su_2020-Burden_W_2020 \| 1,916 \| ,295 \| 6,487 \| ,000 \| ,000 \| \| Burden_Su_2021-Burden_Sp_2020 \| -,827 \| ,295 \| -2,800 \| ,005 \| ,107 \| \| Burden_Su_2021-Burden_A_2020 \| -1,294 \| ,295 \| -4,383 \| ,000 \| ,000 \| \| Burden_Su_2021-Burden_Sp_2021 \| -1,355 \| ,295 \| -4,588 \| ,000 \| ,000 \| \| Burden_Su_2021-Burden_A_2021 \| 1,383 \| ,295 \| 4,683 \| ,000 \| ,000 \| \| Burden_Su_2021-Burden_W_2020 \| -1,766 \| ,295 \| -5,981 \| ,000 \| ,000 \| \| Burden_Sp_2020-Burden_A_2020 \| ,467 \| ,295 \| 1,582 \| ,114 \| 1,000 \| \| Burden_Sp_2020-Burden_Sp_2021 \| -,528 \| ,295 \| -1,788 \| ,074 \| 1,000 \| \| Burden_Sp_2020-Burden_A_2021 \| ,556 \| ,295 \| 1,883 \| ,060 \| 1,000 \| \| Burden_Sp_2020-Burden_W_2020 \| ,939 \| ,295 \| 3,180 \| ,001 \| ,031 \| \| Burden_A_2020-Burden_Sp_2021 \| -,061 \| ,295 \| -,206 \| ,837 \| 1,000 \| \| Burden_A_2020-Burden_A_2021 \| ,089 \| ,295 \| ,301 \| ,764 \| 1,000 \| \| Burden_A_2020-Burden_W_2020 \| -,472 \| ,295 \| -1,598 \| ,110 \| 1,000 \| \| Burden_Sp_2021-Burden_A_2021 \| ,028 \| ,295 \| ,095 \| ,924 \| 1,000 \| \| Burden_Sp_2021-Burden_W_2020 \| ,411 \| ,295 \| 1,392 \| ,164 \| 1,000 \| \| Burden_A_2021-Burden_W_2020 \| -,383 \| ,295 \| -1,297 \| ,194 \| 1,000 \|  \| *Pairwise comparisons, post-hoc Dunn-Bonferroni tests; subjective burden; total sample; work in COVID-19 units* \| \| \| \| \| \| \| --- \| --- \| --- \| --- \| --- \| --- \| \| Sample 1-Sample 2 \| Test Statistics \| Standard Error \| Standard Test Statistics \| Sig. \| Adap. Sig.^a^ \| \| Burden_Su_2020-Burden_Su_2021 \| ,116 \| ,408 \| ,284 \| ,776 \| 1,000 \| \| Burden_Su_2020-Burden_Sp_2020 \| ,875 \| ,408 \| 2,143 \| ,032 \| ,674 \| \| Burden_Su_2020-Burden_A_2020 \| 1,563 \| ,408 \| 3,827 \| ,000 \| ,003 \| \| Burden_Su_2020-Burden_Sp_2021 \| 1,643 \| ,408 \| 4,024 \| ,000 \| ,001 \| \| Burden_Su_2020-Burden_A_2021 \| -1,643 \| ,408 \| -4,024 \| ,000 \| ,001 \| \| Burden_Su_2020-Burden_W_2020 \| 1,848 \| ,408 \| 4,527 \| ,000 \| ,000 \| \| Burden_Su_2021-Burden_Sp_2020 \| -,759 \| ,408 \| -1,859 \| ,063 \| 1,000 \| \| Burden_Su_2021-Burden_A_2020 \| -1,446 \| ,408 \| -3,543 \| ,000 \| ,008 \| \| Burden_Su_2021-Burden_Sp_2021 \| 1,527 \| ,408 \| 3,740 \| ,000 \| ,004 \| \| Burden_Su_2021-Burden_A_2021 \| -1,527 \| ,408 \| -3,740 \| ,000 \| ,004 \| \| Burden_Su_2021-Burden_W_2020 \| -1,732 \| ,408 \| -4,243 \| ,000 \| ,000 \| \| Burden_Sp_2020-Burden_A_2020 \| ,688 \| ,408 \| 1,684 \| ,092 \| 1,000 \| \| Burden_Sp_2020-Burden_Sp_2021 \| ,768 \| ,408 \| 1,881 \| ,060 \| 1,000 \| \| Burden_Sp_2020-Burden_A_2021 \| -,768 \| ,408 \| -1,881 \| ,060 \| 1,000 \| \| Burden_Sp_2020-Burden_W_2020 \| ,973 \| ,408 \| 2,384 \| ,017 \| ,360 \| \| Burden_A_2020-Burden_Sp_2021 \| ,080 \| ,408 \| ,197 \| ,844 \| 1,000 \| \| Burden_A_2020-Burden_A_2021 \| -,080 \| ,408 \| -,197 \| ,844 \| 1,000 \| \| Burden_A_2020-Burden_W_2020 \| -,286 \| ,408 \| -,700 \| ,484 \| 1,000 \| \| Burden_Sp_2021-Burden_A_2021 \| ,000 \| ,408 \| ,000 \| 1,000 \| 1,000 \| \| Burden_Sp_2021-Burden_W_2020 \| -,205 \| ,408 \| -,503 \| ,615 \| 1,000 \| \| Burden_A_2021-Burden_W_2020 \| ,205 \| ,408 \| ,503 \| ,615 \| 1,000 \|   *Pairwise comparisons, post-hoc Dunn-Bonferroni tests; subjective burden; total sample; no work in COVID-19 units* | | | | | |
| --- | --- | --- | --- | --- | --- | --- | --- | --- | --- | --- | --- | --- | --- | --- | --- | --- | --- | --- | --- | --- | --- | --- | --- | --- | --- | --- | --- | --- | --- | --- | --- | --- | --- | --- | --- | --- | --- | --- | --- | --- | --- | --- | --- | --- | --- | --- | --- | --- | --- | --- | --- | --- | --- | --- | --- | --- | --- | --- | --- | --- | --- | --- | --- | --- | --- | --- | --- | --- | --- | --- | --- | --- | --- | --- | --- | --- | --- | --- | --- | --- | --- | --- | --- | --- | --- | --- | --- | --- | --- | --- | --- | --- | --- | --- | --- | --- | --- | --- | --- | --- | --- | --- | --- | --- | --- | --- | --- | --- | --- | --- | --- | --- | --- | --- | --- | --- | --- | --- | --- | --- | --- | --- | --- | --- | --- | --- | --- | --- | --- | --- | --- | --- | --- | --- | --- | --- | --- | --- | --- | --- | --- | --- | --- | --- | --- | --- | --- | --- | --- | --- | --- | --- | --- | --- | --- | --- | --- | --- | --- | --- | --- | --- | --- | --- | --- | --- | --- | --- | --- | --- | --- | --- | --- | --- | --- | --- | --- | --- | --- | --- | --- | --- | --- | --- | --- | --- | --- | --- | --- | --- | --- | --- | --- | --- | --- | --- | --- | --- | --- | --- | --- | --- | --- | --- | --- | --- | --- | --- | --- | --- | --- | --- | --- | --- | --- | --- | --- | --- | --- | --- | --- | --- | --- | --- | --- | --- | --- | --- | --- | --- | --- | --- | --- | --- | --- | --- | --- | --- | --- | --- | --- | --- | --- | --- | --- | --- | --- | --- | --- | --- | --- | --- | --- | --- | --- | --- | --- | --- | --- | --- | --- | --- | --- | --- | --- | --- | --- | --- | --- | --- | --- | --- | --- | --- | --- | --- | --- | --- | --- | --- | --- |
| Sample 1-Sample 2 | Test Statistics | Standard Error | Standard Test Statistics | Sig. | Adap. Sig.^a^ |
| Burden_Su_2020-Burden_Su_2021 | ,186 | ,428 | ,435 | ,663 | 1,000 |
| Burden_Su_2020-Burden_Sp_2020 | 1,088 | ,428 | 2,544 | ,011 | ,230 |
| Burden_Su_2020-Burden_A_2020 | 1,314 | ,428 | 3,071 | ,002 | ,045 |
| Burden_Su_2020-Burden_Sp_2021 | -1,353 | ,428 | -3,163 | ,002 | ,033 |
| Burden_Su_2020-Burden_A_2021 | 1,412 | ,428 | 3,300 | ,001 | ,020 |
| Burden_Su_2020-Burden_W_2020 | 1,990 | ,428 | 4,652 | ,000 | ,000 |
| Burden_Su_2021-Burden_Sp_2020 | -,902 | ,428 | -2,108 | ,035 | ,735 |
| Burden_Su_2021-Burden_A_2020 | -1,127 | ,428 | -2,636 | ,008 | ,176 |
| Burden_Su_2021-Burden_Sp_2021 | -1,167 | ,428 | -2,727 | ,006 | ,134 |
| Burden_Su_2021-Burden_A_2021 | 1,225 | ,428 | 2,865 | ,004 | ,088 |
| Burden_Su_2021-Burden_W_2020 | -1,804 | ,428 | -4,217 | ,000 | ,001 |
| Burden_Sp_2020-Burden_A_2020 | ,225 | ,428 | ,527 | ,598 | 1,000 |
| Burden_Sp_2020-Burden_Sp_2021 | -,265 | ,428 | -,619 | ,536 | 1,000 |
| Burden_Sp_2020-Burden_A_2021 | ,324 | ,428 | ,756 | ,449 | 1,000 |
| Burden_Sp_2020-Burden_W_2020 | ,902 | ,428 | 2,108 | ,035 | ,735 |
| Burden_A_2020-Burden_Sp_2021 | -,039 | ,428 | -,092 | ,927 | 1,000 |
| Burden_A_2020-Burden_A_2021 | ,098 | ,428 | ,229 | ,819 | 1,000 |
| Burden_A_2020-Burden_W_2020 | -,676 | ,428 | -1,581 | ,114 | 1,000 |
| Burden_Sp_2021-Burden_A_2021 | ,059 | ,428 | ,138 | ,891 | 1,000 |
| Burden_Sp_2021-Burden_W_2020 | ,637 | ,428 | 1,490 | ,136 | 1,000 |
| Burden_A_2021-Burden_W_2020 | -,578 | ,428 | -1,352 | ,176 | 1,000 |

| Each row tests the null hypothesis that the distributions in sample 1 and sample 2 are the same. |
| --- |
| Asymptotic significances (two-sided tests) are shown.   1. The significance level is .050. |
